# Editorial: School-based nutrition and physical activity interventions among children and adolescents

**DOI:** 10.3389/fpubh.2025.1585511

**Published:** 2025-03-25

**Authors:** Shooka Mohammadi, Hui Chin Koo

**Affiliations:** ^1^Department of Social and Preventive Medicine, Faculty of Medicine, University of Malaya, Kuala Lumpur, Malaysia; ^2^Department of Bioscience, Faculty of Applied Sciences, Tunku Abdul Rahman University of Management and Technology, Kuala Lumpur, Malaysia

**Keywords:** adolescents, children, school-based, physical activity, nutrition, dietary interventions, dietary patterns

Schools play a crucial role in shaping children's eating habits by providing access to healthy food options, implementing nutrition education programs, and fostering a culture that encourages healthy eating (1). Effective implementation strategies include integrating nutrition education into the curriculum, collaborating with local food suppliers to provide healthier meals, and promoting awareness through school-wide campaigns. By creating a supportive environment, schools can enhance student wellbeing and reinforce positive dietary habits (1). School-based interventions significantly improve students' food choices, encourage healthier eating patterns, and reduce the prevalence of diet-related health issues. The considerable influence of school environments on students' dietary habits highlights the need for healthy canteen interventions as a strategic approach to improve students' nutritional intake (1–3). Effective School-based interventions should focus on enhancing the availability and affordability of nutritious foods, increasing student participation in physical education (PE), and establishing social support systems to promote physical activity (PA) (4).

The increasing prevalence of obesity, sedentary lifestyles, and unhealthy eating habits among children and adolescents is associated with chronic health issues (5–7). Recent data from the World Health Organization indicate that over 39 million children under the age of five were overweight or obese in 2020, underscoring the urgency of preventive interventions. Therefore, promoting healthy eating and sufficient PA among school-aged children is essential. This Research Topic presents the outcomes of 13 school-based studies designed to enhance dietary quality and PA levels in children and adolescents. Among these, four studies focused on nutrition and dietary patterns in children and adolescents, seven examined PA, and two addressed recruitment and outreach within a school-based pediatric obesity intervention, as well as health behaviors and health-related quality of life.

Jha et al. conducted an interventional study to evaluate the impact of a health promotion initiative on the dietary behaviors of Indian adolescents, utilizing the theory of planned behavior (TPB) as a framework. TPB is particularly relevant in this context as it helps explain how attitudes, subjective norms, and perceived behavioral control influence dietary decisions and intentions to adopt healthier eating habits. The study found that the intervention successfully fostered a positive shift in adolescents' intentions to adopt healthier dietary practices. The study highlighted the effectiveness of model-based and construct-oriented intervention strategies in enhancing adolescents' commitment to healthier eating habits (Jha et al.). Devine et al. explored the factors influencing food choices in school canteens, identifying barriers such as convenience, food placement, peer influence and food availability. The study proposed practical, cost-effective strategies, including menu planning, labeling and pricing adjustments, to promote healthier eating habits among secondary school students (Devine et al.).

Sezer et al. investigated the effects of socioeconomic status on the diet quality and snack preferences of adolescents from diverse backgrounds. The findings revealed that adolescents attending public schools had a lower tendency to choose healthy snacks than their peers who attended private schools. This disparity underscores socioeconomic status as a critical determinant of eating behaviors among adolescents. In addition, significant income differences between students attending private and public schools likely contributed to the higher frequency of snack consumption among students attending private schools. Thus, financial resources play a vital role in shaping dietary habits and preferences in this age group (Sezer et al.). A systematic review performed by Ahmed et al. evaluated the effects of the coronavirus disease 2019 (COVID-19) pandemic on school food programs in Canada, focusing on their delivery, adaptability, and resilience. These programs implemented various strategies to address the challenges posed by the pandemic, ensuring that vulnerable students continued to receive nutritious meals. Key initiatives included the distribution of prepared meals, food kits, and gift cards, which effectively enhanced food availability for pupils and their families. The study highlighted the importance of increased collaboration among community members, organizations, and stakeholders as a critical factor in maintaining food delivery and developing new methods for food distribution. However, the study also identified significant challenges related to the sustainability of these programs, particularly operating costs and funding (Ahmed et al.).

Moore, Edmondson et al. conducted a qualitative study to examine PE teachers' perceptions on barriers and facilitators of PA and digital exercise interventions for inactive British adolescents in secondary schools. Using the theoretical domain framework (TDF), the capability, opportunity, motivation, and behavior (COM-B) model, and the behavior change wheel (BCW). The study provided insights into policy functions and behavioral change tools that could enhance PA participation (Moore, Edmondson et al.). Their pre-intervention online survey revealed that while universal barriers affect adolescents' PA, tailored support that considers demographic differences is necessary to effectively promote engagement in PA (Moore, Vernon et al.). Liu Y. et al. proposed a protocol for a 12-week school-based high-intensity interval training (HIIT) intervention to assess its effectiveness on various health and academic outcomes among 12- to 13-year-old students in Ningbo, China (Liu Y. et al.). Peiris et al. planned a protocol for a randomized controlled trial (RCT) to develop and evaluate an in-classroom PA breaks (IcPAB) intervention in Sri Lanka (Peiris et al.). Aly et al. analyzed cross-cultural differences in health-related fitness (HRF) among children from five Mediterranean countries (Italy, Spain, Egypt, Portugal, and Lebanon). Significant variations in HRF were observed across different countries and age groups. The study emphasized the need for culturally tailored PE strategies and public health initiatives to promote balanced fitness development across diverse cultural contexts (Aly et al.).

Wu et al. conducted a network meta-analysis of 66 studies to evaluate the effectiveness of six exercise modalities on various physical fitness indicators within a school-based context. The findings indicated that HIIT was the most effective intervention for reducing body mass index (BMI), enhancing VO2 max, and improving 20-meter sprint performance. Aerobic training was the most effective method for reducing waist circumference (WC). In addition, active video games were recognized as promising options for enhancing countermovement jump and shuttle run performance. Strength training was found to be the most effective for improving standing long jump performance, whereas combined training was rated the highest for reducing body fat percentage and increasing push-up repetitions (Wu et al.). Another network meta-analysis by Hassan et al. evaluated the effectiveness of various school-based obesity prevention initiatives on improving BMI among children and adolescents. The findings revealed that the PA-only intervention was the most effective in enhancing BMI, whereas the multiple-component intervention demonstrated the greatest improvement in BMI z-scores (BMIz). Conversely, the diet and nutrition-only intervention was the least effective for improving BMIz. The study indicated that both PA-only and multiple-component interventions are effective strategies for addressing BMI-related outcomes in school settings (Hassan et al.).

Forseth et al. evaluated two recruitment strategies for a pediatric obesity treatment trial targeting rural families, specifically focusing on school recruitment and participant enrollment rates. The opt-in approach, in which caregivers consented to have their child screened for eligibility, and the screen-first approach, in which all children were screened regardless of prior consent. The findings revealed that schools using the opt-in method were more successful in enrolling at least five families and implementing the intervention. In contrast, the screen-first approach resulted in higher overall participation rates (Forseth et al.). A study performed by Liu Z. H. et al. examined the individual and combined effects of breakfast consumption, sedentary behavior, sleep, and PA on health-related quality of life (HRQoL) among Chinese high school students. It was found that students who used computers for two or more hours daily were more likely to report health issues in areas such as mobility, self-care, and daily activities. Additionally, lower PA levels were correlated with increased feelings of worry and sadness, as well as a lower visual analog scale (VAS) score. Inadequate sleep (defined as <7 h) and skipping breakfast were also associated with poorer HRQoL, particularly concerning pain, discomfort, and emotional wellbeing (Liu Z. H. et al.).

In summary, the articles in this Research Topic have provided a foundational framework for developing school-based interventions to improve nutrition and PA among adolescents and children. Further studies should focus on the long-term impact of these interventions, as well as strategies for scaling them up at a national or international level. By addressing key behavioral, environmental, and socioeconomic factors, future interventions can be designed to maximize effectiveness and sustainability. This Research Topic underscores the need for continued public support and policy initiatives to enhance wellbeing and quality of life of school-aged students.
